# Deep Learning-Based Temporal Gait Analysis Using a Smartphone IMU in Older Adults with and Without Non-Specific Low Back Pain

**DOI:** 10.3390/bioengineering13080924

**Published:** 2026-08-14

**Authors:** Gerome Vivar, Shivam Singh, Tobias Bea, Christian Saal, Victor Munoz-Martel, Lutz Schega

**Affiliations:** Health and Physical Activity, Otto von Guericke University Magdeburg, 39104 Magdeburg, Germany; shivam.singh@ovgu.de (S.S.); lutz.schega@ovgu.de (L.S.)

**Keywords:** deep learning, gait analysis, gait event detection, non-specific low back pain, smartphone IMU, transfer learning

## Abstract

Accurate gait event detection using inertial measurement units (IMUs) is essential for temporal gait analysis, but frame-level detection is challenged by sparse initial contact (IC) and foot-off (FO) events. This study evaluated recurrent neural network architectures and training strategies for simultaneous IC and FO detection using a single shank-mounted smartphone IMU. The internal dataset included 28 healthy older adults and 18 individuals with non-specific low back pain (NSLBP). Temporal label expansion substantially improved validation performance for gated recurrent unit (GRU) and long short-term memory models, whereas point-label and class-weighted training performed poorly. The selected label-expanded GRU (LE-GRU) achieved $F_{1}$ scores above 0.95 for both events and mean absolute temporal errors below 12 ms on held-out internal test folds, with high performance in both cohorts. On an external dataset with different sensor and acquisition characteristics, high performance required full-network fine-tuning, indicating the need for adaptation across datasets. Stance phase and stride time calculated from LE-GRU-predicted events showed high agreement with reference-derived values, with Lin’s concordance correlation coefficients from 0.980 to 0.994. These findings demonstrate that temporal label expansion enables accurate GRU-based gait event detection and temporal gait analysis from data collected with a single smartphone IMU.

## 1. Introduction

Gait analysis is essential in the clinical assessment of gait disorders, monitoring disease progression, and evaluating rehabilitation outcomes [1,2,3]. A core element of this process is the accurate identification of key gait events, especially initial contact (IC), where the foot makes its initial ground contact marking the start of the stance phase, and foot-off (FO), marking the moment the foot lifts from the ground to initiate the swing phase [4,5]. These events define the stance and swing phases of the gait cycle and enable the calculation of temporal gait parameters such as stance phase, swing phase, and stride time [6]. Such temporal gait parameters are commonly used as outcome measures in gait rehabilitation studies involving older adults [7].

The reference standard for detecting gait events is force-plate measurement of ground reaction forces [4,5,8]. However, this approach is restricted by a limited capture area and high cost, and is prohibitive for mobile applications [9]. As a result, clinical practice often relies on manual visual observation of movement, a qualitative method that is exceptionally time-intensive and prone to subjective assessments [1,3,10].

To overcome these constraints, the increasing availability of mobile devices equipped with inertial measurement units (IMUs) provides a practical, low-cost alternative for collecting temporal gait kinematics [11,12]. Early IMU-based methods relied on heuristic rules (e.g., threshold- and peak-based algorithms) to detect gait events; however, their performance can be limited by variability in gait patterns, sensor placement, and acquisition conditions [8,13,14]. This limitation is particularly problematic in conditions such as non-specific low back pain (NSLBP) and neurodegenerative diseases, where changes in temporal gait kinematics may be robust features for clinical evaluations.

Low-back pain (LBP) ranks among the leading causes of disability globally [15,16] and is projected to increase in burden by 2050 [16] due to the growth and aging of the global population. Within this population, individuals with NSLBP may adopt altered gait strategies including reduced gait speed, cadence, and step length [17]. Consequently, a recent meta-analysis on NSLBP suggested that further high-quality studies are needed to improve evidence quality and to better understand which factors contribute to these changes in temporal gait kinematics [17]. At the same time, the review also suggested that IMU-based systems should be further explored as a feasible alternative to marker-based optoelectronic devices. This highlights the need for accurate automated gait event detection using IMU sensors to improve result consistency and strengthen evidence quality.

To address the limitations of heuristic-based gait event detection, recent studies have increasingly adopted deep learning (DL) approaches. Recurrent neural networks (RNN), including long short-term memory (LSTM) and gated recurrent unit (GRU) models, are particularly suitable for modeling temporal dependencies in gait signals [18,19]. Their applicability to pathological gait has been demonstrated in pediatric and orthopedic cohorts, with reported detection accuracies exceeding 98% [8,20]. However, these studies commonly relied on laboratory-grade measurement systems, leaving the application of recurrent models to single-sensor smartphone IMU data insufficiently explored.

In the context of wearable sensors, recent studies have shown the applicability of these DL approaches. Convolutional Neural Networks (CNNs) and hybrid models (CNN-RNN) have achieved high detection accuracies using waist-mounted sensors [21,22]. However, waist-mounted sensors may capture less detailed lower-limb dynamics than sensors positioned at the shank or foot, and are highly susceptible to motion artifacts induced by hand movements. While adaptive algorithms have been applied to foot-mounted sensors in neurological cohorts [11,14], foot-mounted sensors may be less practical for daily monitoring than shank-mounted sensors. Notably, recent evidence indicates that DL models can be robust to sensor location, achieving high detection accuracy (*F*_1_-score > 96%) using a single shank-mounted IMU within diverse neurological cohorts [23]. More recently, hybrid CNN–BiLSTM–Transformer architectures have also achieved high performance in wearable IMU-based gait segmentation, with performance comparable to recurrent models alone [24].

Despite these advancements, four important gaps remain. First, IC and FO are temporally sparse relative to the dominant non-event (NE) class, and although related target-modification strategies have been explored in other sequence-learning applications [25,26], the effectiveness of temporal label expansion for learning these sparse events has not been systematically evaluated for gait event detection. Second, participant-independent evaluation of learning-based gait event detection in older adults with NSLBP using a single smartphone IMU remains limited. Although a previous proof-of-concept study applied LSTM networks to gait event estimation in chronic low back pain [27], validation in a larger older cohort is still needed. Third, high event-detection performance does not necessarily ensure accurate derived temporal gait parameters, and the propagation of event-timing errors into stance phase and stride time requires further evaluation. Fourth, the transportability of IMU-based deep learning models across datasets with different sensor and acquisition characteristics remains insufficiently understood.

To address these gaps, this study aims to:

1. validate IC and FO detection on unseen participants using a single shank-mounted smartphone IMU in older adults with and without NSLBP;
2. evaluate whether temporal label expansion improves learning of sparse frame-level gait events;
3. descriptively compare detection performance between individuals with NSLBP and healthy older adults;
4. quantify the impact of event-detection errors on derived temporal gait parameters, specifically stance phase and stride time; and
5. assess model transportability to an external dataset with different sensor and acquisition characteristics.

## 2. Materials and Methods

### 2.1. Data Acquisition and Cohorts

This work utilized two datasets, one internally acquired and one publicly available. The internal dataset involved two primary cohorts (Table 1), totaling 46 participants: 28 control subjects and 18 individuals with NSLBP (self-reported pain score: 4.6±1.3).

Participants with NSLBP were older than 50 years. Exclusion criteria included lower-extremity orthopedic or muscular disorders, lower-extremity malalignment, and lower-extremity injury or surgery within the preceding six months. All participants provided written informed consent. The study was approved by the Ethics Committee of Otto von Guericke University Magdeburg (approval number 182/18) (German Clinical Trial Register, ID: DRKS00021696, https://drks.de/search/de/trial/DRKS00021696/details (accessed on 10 August 2026)). Participants walked back and forth on a 6 m flat surface for 2 min at a self-selected pace, yielding 5911 strides in the internal dataset. Data were collected using an iPhone 15 Pro (iOS 18.6.2), sampling accelerometer and gyroscope data at 100 Hz. The device was securely attached to the lateral aspect of the right shank, approximately 1 cm proximal to the right tibial marker used by the Vicon motion-capture system (Vicon Motion Systems Ltd., Oxford, UK). The device was mounted on the shank with the z-axis oriented laterally and the x-axis anteriorly. Vicon data were not used for gait-event annotation or analysis.

The external dataset [28] comprises motion capture, 3-D biomechanical, and wearable sensor data collected from 22 healthy adults (21±3.4 years). Only the IMU data (accelerometer and gyroscope) recorded from the right shank during the level-ground walking trials were utilized. This dataset was downsampled to 100 Hz to match the sampling rate of the internal dataset used to train the GRU models. Reference IC and FO annotations were provided within the external dataset.

### 2.2. Data Processing and Labeling

Raw linear acceleration and angular velocity signals were low-pass filtered using a fifth-order Butterworth filter with a cutoff frequency of 5 Hz to reduce noise. Reference annotations for IC and FO were generated using an algorithm-assisted labeling workflow. Candidate events were first initialized using the cumulative mediolateral shank angular velocity (CSAV) method [29] and were subsequently reviewed and manually corrected according to predefined IC and FO criteria based on the negative and positive zero-crossings of the shank angular velocity signal. The resulting manually reviewed annotations served as the reference labels for model training, validation, and testing.

After participant-level data splitting, the processed signals and corresponding labels were segmented into windows of 256 samples. Training windows were generated with 50% overlap, whereas validation and test windows were generated without overlap to match the final evaluation setting. This resulted in a three-dimensional input tensor of shape B×L×D, where *B* denotes the number of windows, L=256 the sequence length, and D=6 the three accelerometer and three gyroscope channels. No signal-level data augmentation was explored, as physically plausible transformations of orientation-dependent IMU signals require careful consideration and were beyond the scope of this work.

An initial downsampling step was performed on the external dataset before pre-processing to match the sampling rate of the internal dataset. Data downsampling was performed by a factor of two (from 200 Hz to 100 Hz) using the decimate function [30], which applies a zero-phase Finite Impulse Response filter to the six IMU channels (three gyroscope and three accelerometer axes) to prevent aliasing prior to reducing the sample rate. For the reference labels (IC and FO) in the external dataset, the original label indices were mapped to the downsampled sequence to maintain temporal correspondence. This was performed by dividing the original label index by the downsampling factor (2) and rounding to the nearest integer index in the downsampled sequence.

### 2.3. Deep Learning Model Architecture

#### 2.3.1. Recurrent Neural Networks

**Figure 1:**
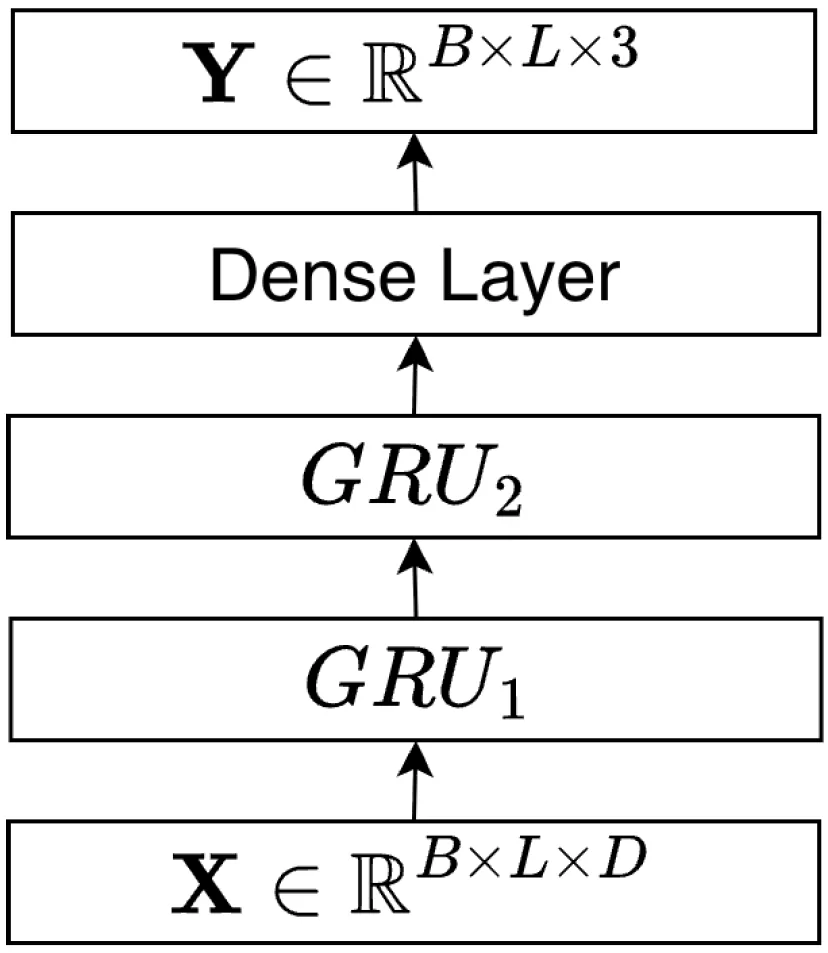
Illustration of the GRU architecture for three-class gait event detection.

The GRU model comprised two stacked GRU layers [19] (Figure 1), each containing 64 hidden units and utilizing a hyperbolic tangent activation function. The LSTM model used the same architecture, except that the GRU layers were replaced by LSTM layers. Both architectures used 64 hidden units per recurrent layer, a dropout probability of 0.5, and an identical three-class output layer. A time-distributed fully connected layer with three output units, followed by log-softmax activation, produced frame-level probabilities for the non-event (NE), IC, and FO classes.

#### 2.3.2. Temporal Label Expansion

IC and FO were originally represented as single-frame labels. During training, each event label was symmetrically expanded by ±10, ±20, ±40, or ±80 ms around its reference frame. At the 100 Hz sampling rate, these expansion widths corresponded to ±1, ±2, ±4, and ±8 samples and were selected to evaluate progressively wider temporal neighborhoods around each event. The final configuration was selected empirically based on validation performance. Label expansion was applied only to the training labels. Validation and test labels remained single-frame events. Point-label training and class-weighted negative log-likelihood loss were evaluated as comparison conditions.

A GRU model trained using temporal label expansion is hereafter referred to as the label-expanded GRU (LE-GRU).

#### 2.3.3. Prediction Post-Processing

The model generated a frame-level sequence with three classes: NE, IC, and FO. Because temporal label expansion resulted in consecutive frames being assigned to the same gait event, each contiguous run of identical non-zero predictions was reduced to a single point event. The temporal midpoint of each run was selected as the predicted IC or FO timestamp. The resulting point-event sequence was used for event matching, temporal-error calculation, and downstream temporal gait analysis. For runs containing an even number of frames, the earlier of the two central frames was selected.

### 2.4. Model Training and Evaluation

Model training and evaluation were conducted using five-fold stratified participant-independent cross-validation (CV) with an internal stratified validation split. Participants were first partitioned into five stratified folds according to cohort label (healthy or NSLBP). In each CV run, one fold was held out as the test set, such that each participant served as test data exactly once across the five runs. The remaining four folds were further split into training and validation subsets using a stratified hold-out split, with 75% used for training and 25% used for validation. This resulted in approximately 60%, 20%, and 20% of the full dataset being used for training, validation, and testing, respectively. For a representative CV fold, the training, validation, and test subsets contained 3441/3416, 1165/1156, and 1347/1339 IC/FO events, respectively. The validation set was used for checkpoint selection and optimization decisions, whereas the test set was held out for final performance evaluation. Data from the same participant were not shared across the training, validation, and test subsets.

Model weights were optimized using the Adam optimization algorithm [31] (initial learning rate $\alpha =1\times 10^{-3}$) with a batch size of 256 to minimize the negative log-likelihood loss. Training proceeded for a maximum of 250 epochs, with the final models selected based on the highest $F_{1}$ score on the dedicated validation set. No early stopping was applied. Furthermore, to enhance convergence stability, the learning rate was reduced by half when the validation loss did not improve after five epochs. Training was implemented using PyTorch (v2.9.0). Training was performed on an NVIDIA A30 GPU and required approximately 4.6 min per CV fold. Mean inference time for a single 256×6 input window was 0.711 ms (SD = 0.010 ms), measured over 100 runs after 20 warm-up runs on the same GPU. The final LE-GRU contained 38,979 trainable parameters, corresponding to approximately 0.15 MB of model weights. All experiments used a random seed of 42 for reproducibility.

Performance was evaluated separately for IC and FO using the $F_{1}$ score and mean absolute temporal error. Event matching was performed independently for each event type using one-to-one matching. A prediction was classified as a true positive when it occurred within ±30 ms of an unmatched reference event, consistent with the tolerance window used in [32]. Unmatched predictions were classified as false positives, and unmatched reference events were classified as false negatives. For temporal-error analysis, each reference event was matched to the nearest unmatched prediction within ±200 ms, and the mean absolute time difference was calculated. IC-FO macro-$F_{1}$ was defined as the unweighted mean of the IC and FO $F_{1}$ scores.

For architecture and training-strategy selection, GRU and LSTM models were trained using identical participant splits, hyperparameters, and evaluation procedures. Each architecture was evaluated using the original point labels, class-weighted negative log-likelihood loss, and temporal label expansion. Model and training-strategy selection was performed exclusively using validation-set IC–FO macro-$F_{1}$ scores; the outer-fold test sets were reserved for final performance evaluation.

For external-dataset adaptation, participants were divided at the participant level into fine-tuning and held-out test subsets. The fine-tuning subset contained 50% of the external participants, while the remaining 50% were reserved for evaluation. Three strategies were compared: zero-shot evaluation, fine-tuning of the final classification layer, and fine-tuning of the full network.

### 2.5. Temporal Gait-Parameter Calculation

Stride time was calculated as the interval between consecutive IC events:

(1) $$T_{\mathrm{stride},i}=t_{\mathrm{IC},i+1}-t_{\mathrm{IC},i}.$$

Stance phase was calculated as the percentage of the gait cycle between IC and the subsequent FO:

(2) $$P_{\mathrm{stance},i}=100\frac{t_{\mathrm{FO},i}-t_{\mathrm{IC},i}}{t_{\mathrm{IC},i+1}-t_{\mathrm{IC},i}}.$$

Both parameters were calculated independently using LE-GRU-predicted and reference gait events.

### 2.6. Statistical Analysis

Agreement between temporal gait parameters calculated from LE-GRU-predicted and reference gait events was evaluated using Lin’s concordance correlation coefficient (CCC) [33], root mean square error (RMSE), mean absolute error (MAE), and Bland–Altman analysis [34]. For Bland–Altman analysis, differences were calculated as the LE-GRU-derived value minus the reference-derived value. Mean bias and the 95% limits of agreement were calculated as the mean difference ±1.96 standard deviations. The 95% confidence intervals for Lin’s CCC were estimated using a paired bias-corrected and accelerated (BCa) bootstrap with 1000 resamples. Analyses were performed separately for the internal and external datasets. Comparisons of gait-event detection performance between the healthy and NSLBP cohorts were descriptive.

## 3. Results

### 3.1. Architecture and Training-Strategy Comparison

**Figure 2:**
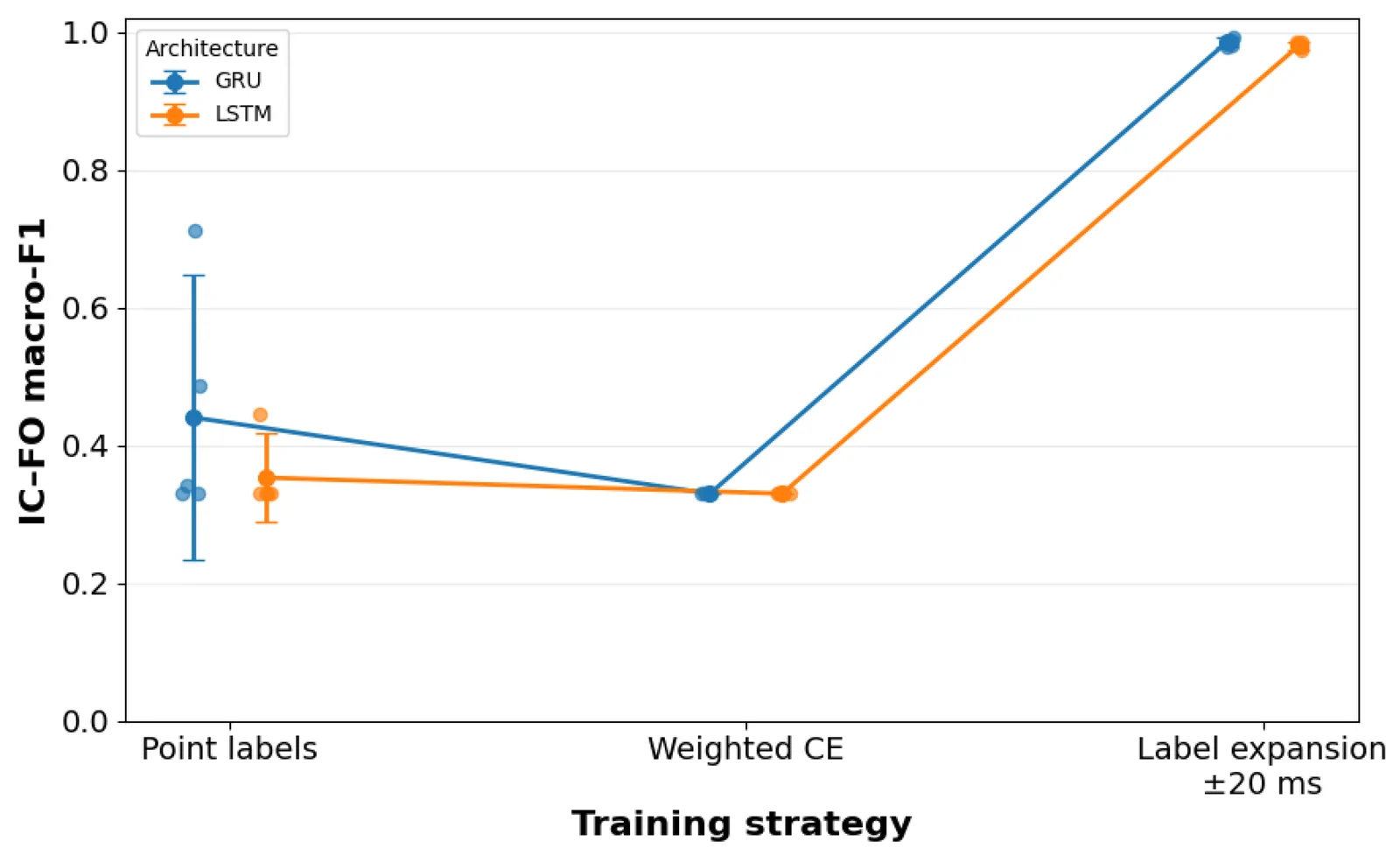
Validation IC–FO macro-$F_{1}$ scores for GRU and LSTM models using point labels, class-weighted loss, and temporal label expansion of ±20 ms. Label expansion substantially improved performance for both architectures. Points represent individual CV folds, and error bars indicate mean ± standard deviation.

GRU and LSTM models were compared using point labels, class-weighted loss, and temporal label expansion of ±20 ms. As shown in Figure 2, point-label and class-weighted training produced low validation performance for both architectures. Label expansion increased the IC–FO macro-$F_{1}$ score to approximately 0.98 for both models, demonstrating that label expansion was the key factor enabling effective learning of the sparse gait events. The GRU model was selected because it provided the best trade-off between validation performance and model complexity.

### 3.2. Temporal Label-Expansion Analysis and Final Model Selection

**Figure 3:**
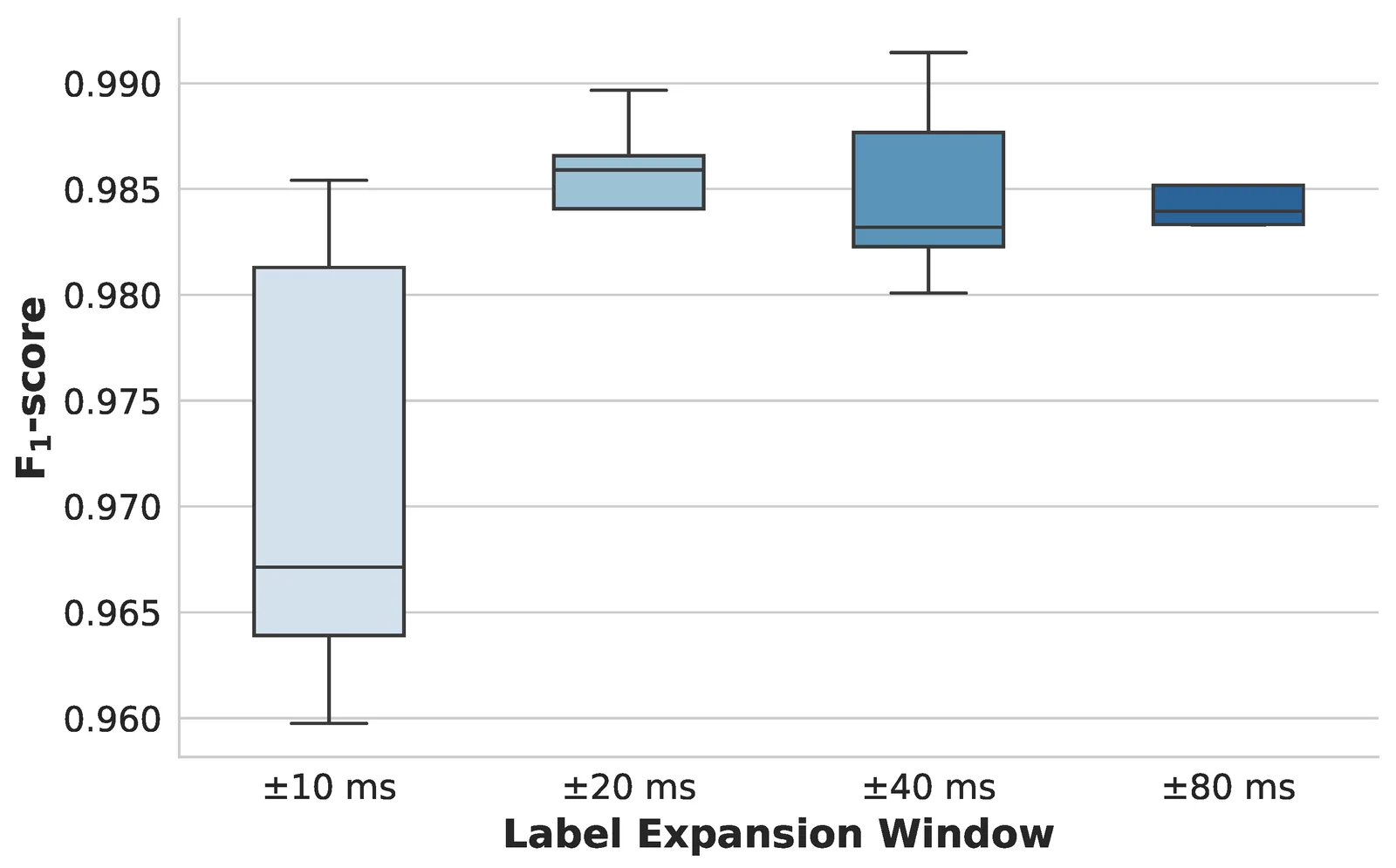
Gait event detection performance on the validation set after five-fold CV. The model trained without label expansion (±0 ms) is excluded from the plot for brevity; see Table 2.

Following selection of the GRU architecture, the effect of temporal label-expansion width was evaluated using expansion intervals of ±0, ±10, ±20, ±40, and ±80 ms. Without label expansion, the model did not learn the sparse IC and FO event classes effectively, resulting in substantially lower validation performance (Table 2 and Figure 3).

Expanding the event labels by at least ±10 ms produced a substantial improvement in the IC–FO macro-$F_{1}$ score (Table 2 and Figure 3). Performance was similar for expansion widths between ±20 ms and ±80 ms. An expansion width of ±20 ms was selected because it provided near-maximal validation performance while applying the smallest temporal expansion among the best-performing configurations. The ±20 ms LE-GRU configuration was used for the remaining experiments.

### 3.3. Detection Performance on the Internal Test Set

**Figure 4:**
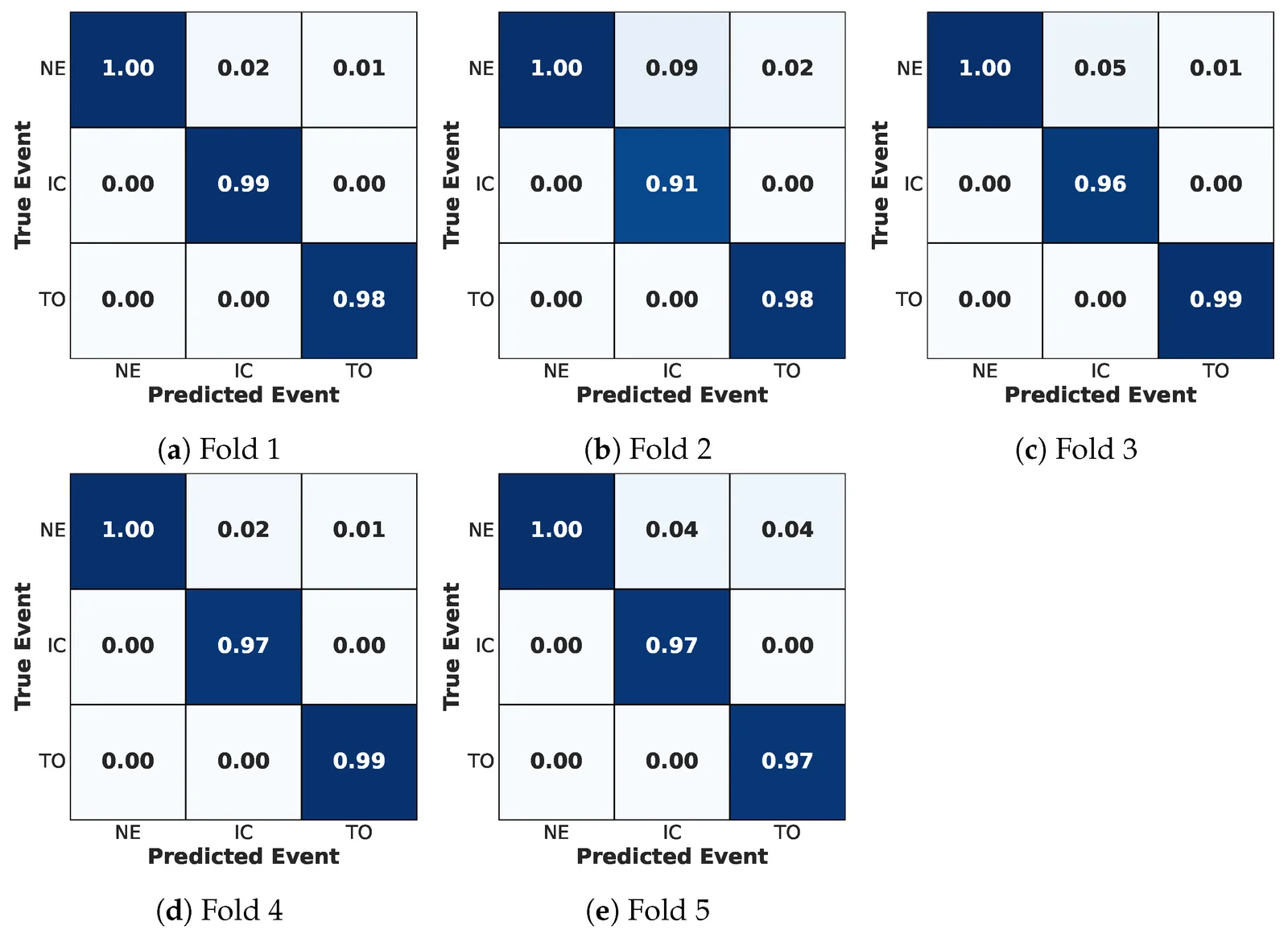
Detection performance on every outer-fold test set after five-fold CV.

Using the validation-selected model from each CV fold, performance was evaluated on the corresponding held-out test set (Figure 4). Across the five folds, mean sensitivity was 96% for IC and 98% for FO. Performance remained consistently high across event classes and folds, although IC sensitivity was slightly lower in one fold. Overall, IC and FO sensitivity ranged from 91% to 99%.

### 3.4. Comparative Performance on Internal Cohorts

**Figure 5:**
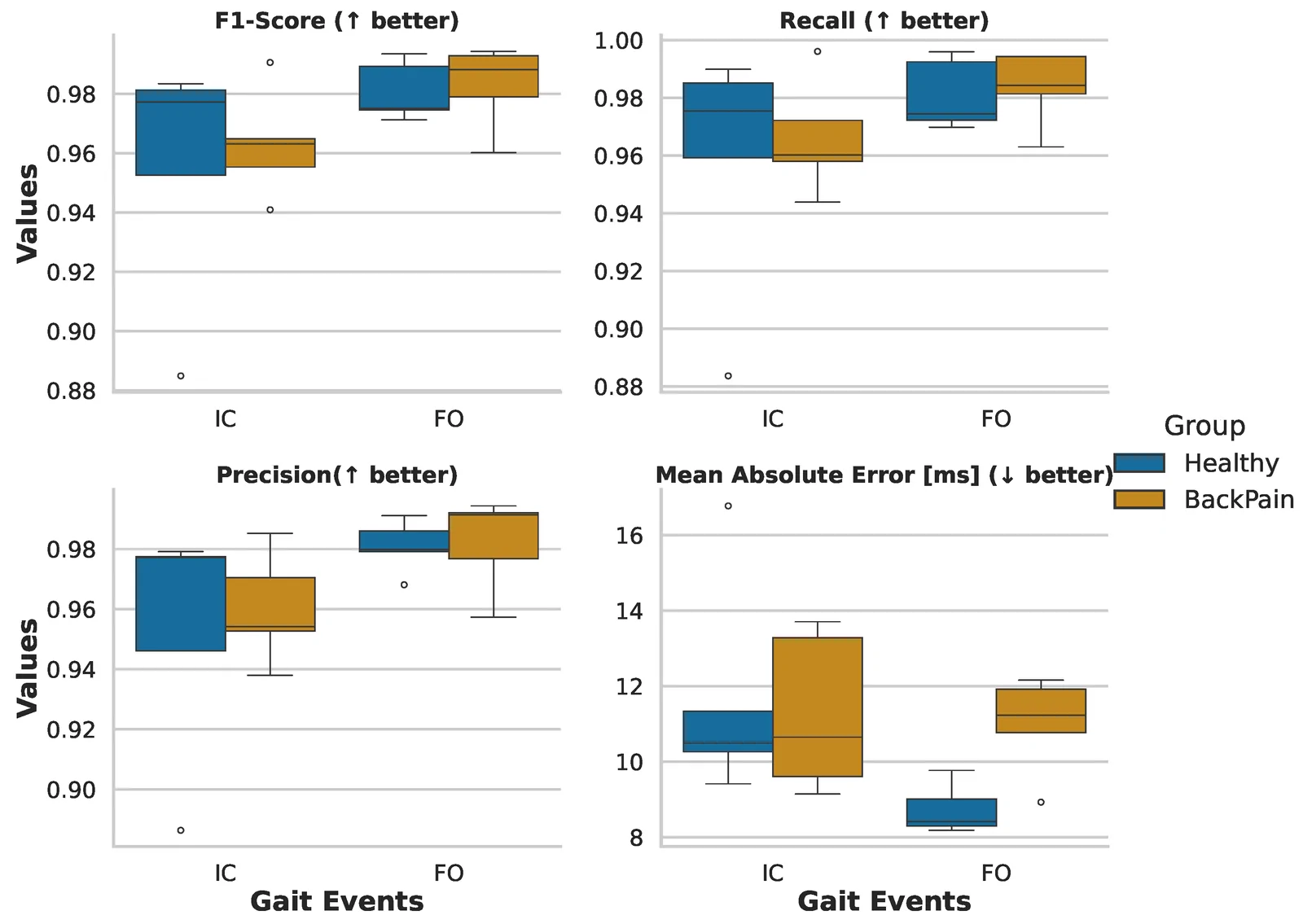
Overall test set performance between the healthy and back pain groups (five-fold CV).

The proposed model was further analyzed on the internal test set to compare performance metrics between the healthy and NSLBP cohorts. As shown in Figure 5, the proposed model demonstrated high performance in detecting gait events (IC and FO) in both healthy control and NSLBP cohorts. Detection performance, quantified by $F_{1}$ score, precision, and recall, yielded median scores consistently above 95% across all events and groups, with no substantial difference observed between the cohorts (as shown in Figure 5). Temporal accuracy was also high, with median MAE values well under 12 ms overall. Specifically, the healthy group achieved high $F_{1}$ scores (0.977 for IC and 0.975 for FO) and low MAE (10.5 ms for IC and 8.4 ms for FO). Performance also remained high in the NSLBP cohort, with median $F_{1}$ scores of 0.963 for IC and 0.988 for FO and median MAEs of approximately 10.6 ms and 11.2 ms, respectively (Figure 5).

### 3.5. Generalization Performance on the External Dataset

**Figure 6:**
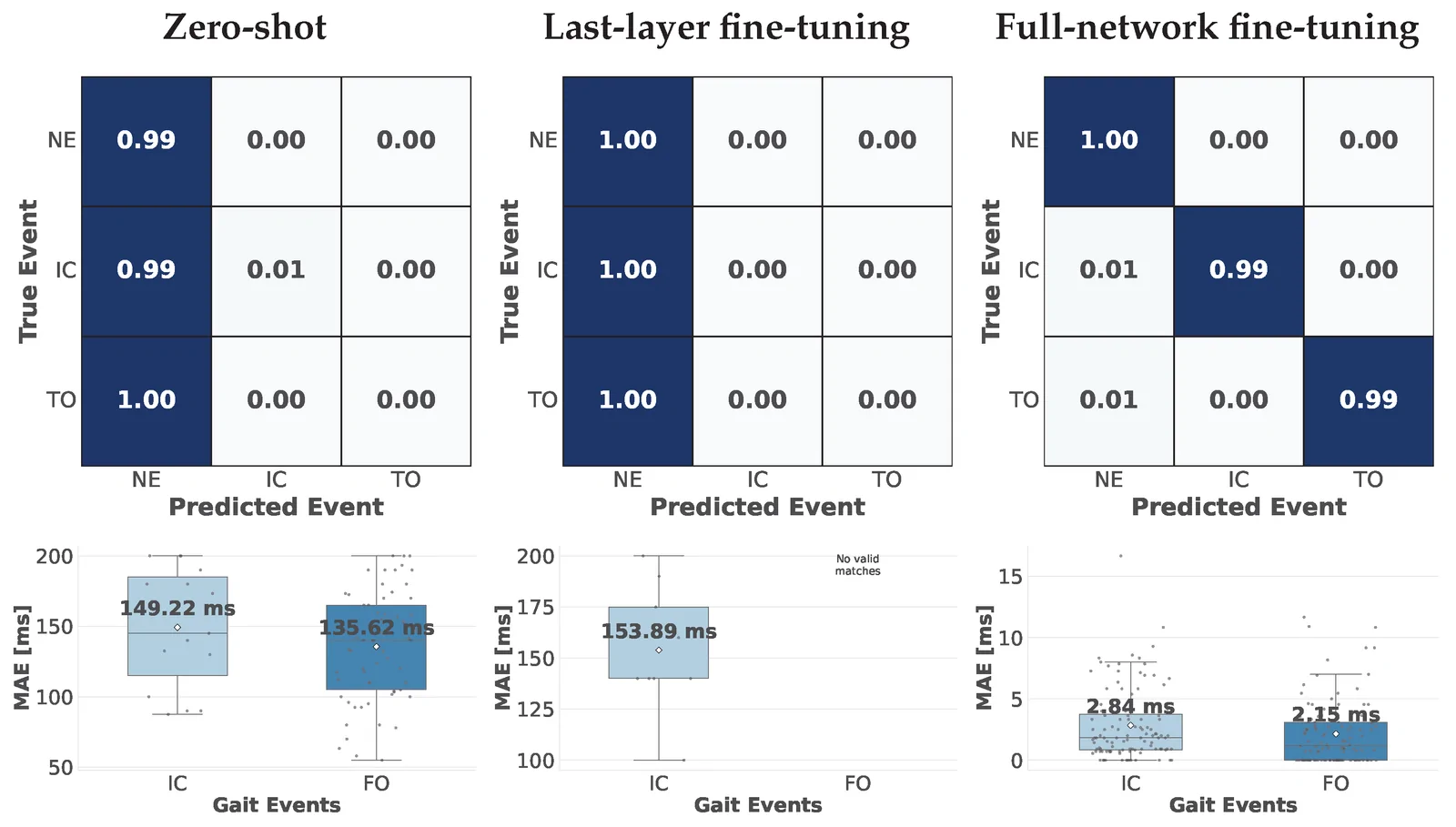
Detection performance and temporal error on the external dataset under zero-shot evaluation, last-layer fine-tuning, and full-network fine-tuning. Diamonds and numerical labels indicate mean temporal errors. No FO temporal error was calculated for last-layer fine-tuning because no valid FO matches were obtained.

The external dataset [28] was used to evaluate three adaptation strategies: zero-shot evaluation, last-layer fine-tuning, and full-network fine-tuning. Zero-shot evaluation and last-layer fine-tuning failed to generalize, with most frames classified as NE, resulting in poor IC and FO detection and temporal errors of approximately 150 ms (Figure 6). Full-network fine-tuning achieved substantially higher performance, with IC and FO recall of approximately 0.99 and MAE below 5 ms.

### 3.6. Agreement of Derived Temporal Gait Parameters

Temporal gait parameters calculated from the LE-GRU-predicted events showed high agreement with those calculated from the reference events (Table 3). Across the internal and external datasets, Lin’s CCC ranged from 0.980 to 0.994. The stance-phase MAE remained below 0.3 percentage points, while stride-time MAE ranged from 8 ms to 17 ms. These results indicate that gait-event detection errors had only a limited effect on the derived temporal gait parameters.

## 4. Discussion

The primary goal of this study was to develop a deep learning approach for gait event detection using a single smartphone IMU and to evaluate its performance in healthy older adults and individuals with NSLBP. The architecture and training-strategy comparison showed that temporal label expansion was the key factor enabling effective learning of the sparse gait events. The selected LE-GRU achieved $F_{1}$ scores above 0.95 for IC and FO and temporal errors below 12 ms on the held-out internal test folds. Descriptively, performance also remained high in both internal cohorts. On the external dataset, high performance was achieved only after full-network fine-tuning, indicating that adaptation was required to address the domain shift. Furthermore, stance phase and stride time derived from LE-GRU predictions showed high agreement with the reference-derived parameters.

Temporal label expansion increased the amount of event-class supervision while retaining the original point-event annotations for validation and testing. Unlike previous label smoothing approaches, which soften class-probability targets at each time point [25], and temporal label smoothing, which varies soft target distributions according to temporal proximity to an event [26], temporal label expansion retains hard categorical labels and extends the event class to neighboring time samples during training. The similar improvement observed for both GRU and LSTM models suggests that performance was primarily determined by the representation of the sparse training labels rather than by the recurrent architecture. The GRU was retained because it achieved comparable performance with lower model complexity.

### 4.1. Comparison to Prior Gait Event Detection Approaches

Prior IMU-based approaches to gait event detection utilized either thresholding or conventional machine learning (ML) techniques. While threshold-based methods are computationally inexpensive compared to learning-based approaches, their high sensitivity to sensor placement and individual pathology could limit their clinical utility. Conventional ML models, such as hidden Markov models [11] and Support Vector Machines [13], reported comparable performance in healthy cohorts ($F_{1}$ scores up to 0.975) in comparison to the current study. However, these approaches required extensive feature engineering and demonstrated low generalization performance in non-healthy groups. Specifically, one study reported a sensitivity of 75% for IC detection [11].

Recent DL-based approaches have reported high gait-event detection performance, although direct comparisons are limited by differences in datasets, sensing modalities, event definitions, and evaluation protocols. Hence, a direct comparison to the current proposed approach is limited due to varying evaluation protocols and sensing modalities. First, studies based on non-IMU data, such as marker-based motion capture or force plates [8,20,32], are not directly comparable. These methods rely on a fundamentally different, laboratory-grade source of signal. Consequently, their reported performance does not directly establish feasibility for portable smartphone-based monitoring.

Second, studies based on IMU data, such as those using DL approaches like CNN/LSTM utilized for automated gait event detection [21], reported high $F_{1}$ scores (≈99%). However, these results frequently relied on tolerance windows substantially broader than the strict ±30 ms criterion used in the current study. Similarly, a hybrid CNN–BiLSTM–Transformer model using multiple IMUs achieved an $F_{1}$ score of 98.16% for gait segmentation across normal and simulated abnormal gait patterns [24]. However, it classified heel, toe, and full-foot movement states rather than discrete IC and FO events. Finally, while the majority of high-performing models use DL, the use of accuracy as the primary metric, as in other studies (e.g., [27]), can be suboptimal. Since gait event detection involves a highly class-imbalanced context, accuracy can inflate detection performance, limiting direct comparability to the $F_{1}$ score metrics presented in the current study.

Among existing IMU-based studies, ref. [23] provides the closest comparison, as their work included a shank-mounted IMU, similar to the configuration utilized in the current study. Their Temporal Convolutional Network (TCN) achieved high detection performance (IC *F*_1_-scores = 96%, FO *F*_1_-scores = 97%) and low temporal errors (MAE≈5ms). However, the study relied on a laboratory-grade IMU and its tolerance window for detecting TP was not specified, limiting direct comparison. Nonetheless, the current proposed LE-GRU approach shows comparable performance (*F*_1_-score ≥ 0.95 for IC/FO, Mean Absolute Error<12 ms) despite using smartphone-based IMU. This highlights the effectiveness of learning-based approaches, such as neural networks, for supervised gait event detection tasks.

Compared with recent hybrid architectures, the proposed LE-GRU uses a simpler recurrent neural network architecture and a single shank-mounted smartphone IMU for direct IC and FO detection, while achieving comparable detection performance. Overall, IMU-based gait event detection performance varies with sensor placement, hardware, and evaluation strategy, as summarized in Table 4. Within this context, the LE-GRU approach achieved competitive detection accuracy and temporal precision using data from a single smartphone-based IMU. However, further validation under free-living conditions is essential.

### 4.2. Translational Utility for Clinical Gait Analysis

Detection performance was descriptively similar in the NSLBP and healthy control cohorts (Figure 5). Agreement analysis showed that event-timing errors of approximately 10 ms had only a limited effect on the derived gait parameters, with stance-phase MAE below 0.3 percentage points and stride-time MAE between 8 ms and 17 ms. Lin’s CCC ranged from 0.980 to 0.994 across stance phase and stride time in the internal and external datasets (Table 3). These findings support the use of LE-GRU-derived events for subsequent temporal gait analysis. However, the present analysis evaluated measurement performance rather than the diagnostic or prognostic utility of the derived gait parameters.

### 4.3. Cross-Dataset Transportability and Full-Network Adaptation

The poor performance of zero-shot evaluation and last-layer fine-tuning indicates a substantial domain shift between the internal and external datasets. Differences in sensor hardware, mounting, sampling characteristics, acquisition procedures, and participant populations could not be addressed by adapting only the classification layer. Full-network fine-tuning substantially improved detection and temporal accuracy, suggesting that adaptation of the learned feature representations was required. Because this procedure used labeled data from 50% of the external dataset, these findings demonstrate model transportability after adaptation rather than direct zero-shot generalization.

### 4.4. Limitations and Future Directions

The internal dataset was limited to older healthy adults and individuals with NSLBP during steady-state level-ground walking, whereas the external dataset included younger healthy adults. Validation across additional clinical populations, walking speeds, and locomotion conditions, including ramps and stairs, is therefore needed. In practical use, variations in smartphone position or orientation on the shank may affect IMU signals and model performance, warranting further evaluation. Further, the reported inference time was measured on an NVIDIA A30 GPU and should not be interpreted as direct smartphone performance. Real-time on-device benchmarking remains future work.

External validation was also limited to one dataset, and full-network fine-tuning required labeled data from 50% of that dataset. The amount of labeled data required for successful adaptation was not systematically evaluated in the present study and remains to be established. Future work should investigate more sample-efficient adaptation, sensor-location-agnostic models, and validation across additional devices and populations. Approaches such as domain adaptation, self-supervised learning, and few-shot learning could also be explored to improve cross-dataset generalization. The approach could also be extended toward direct estimation of continuous gait parameters rather than sequential event detection followed by parameter calculation. Further, reference events were established using a standardized CSAV-assisted manual annotation workflow. Future studies may further confirm these findings against an independent biomechanical reference.

## 5. Conclusions

This study demonstrates that temporal label expansion enables effective GRU-based detection of IC and FO from a single shank-mounted smartphone IMU. The selected LE-GRU achieved high detection performance and low temporal error in healthy older adults and individuals with NSLBP. Stance phase and stride time derived from the predicted events also showed high agreement with reference-derived values. Performance on the external dataset required full-network fine-tuning, highlighting the importance of adaptation when transferring IMU-based models across datasets. Overall, the LE-GRU approach supports accurate temporal gait analysis using data acquired with a single-smartphone IMU setup.

## Figures and Tables

**Table 1:** Demographic characteristics of the study participants.

	Healthy (*n* = 28)	NSLBP (*n* = 18)	Overall (*n* = 46)
Age (years)	69.5 ± 7.0	68.8 ± 6.2	69.2 ± 6.7
Height (cm)	160.0 ± 23.1	164.8 ± 7.9	161.9 ± 18.7
Weight (kg)	74.3 ± 21.2	74.3 ± 13.5	74.3 ± 18.4
Male (*n*, %)	6 (21.4%)	4 (22.2%)	10 (21.7%)
Female (*n*, %)	22 (78.6%)	14 (77.8%)	36 (78.3%)

**Table 2:** Gait event detection performance on the validation set when expanding the reference labels during training.

Label Expansion	CV Folds	Mean ± Std. Dev.
±0 ms	5	0.481 ± 0.176
±10 ms	5	0.971 ± 0.011
±20 ms	5	0.983 ± 0.008
±40 ms	5	0.985 ± 0.005
±80 ms	5	0.985 ± 0.002

**Table 3:** Agreement between temporal gait parameters calculated from LE-GRU-predicted and reference gait events.

Data	Param.	CCC [95% CI]	RMSE	MAE	Bias	95% LoA
Internal	Stance phase (pp)	0.980 [0.953–0.998]	0.425	0.260	−0.26	[−0.95, 0.43]
Internal	Stride time (ms)	0.994 [0.974–0.998]	9	8	−10	[−20, 10]
External	Stance phase (pp)	0.984 [0.908–1.0]	0.344	0.120	−0.12	[−0.78, 0.55]
External	Stride time (ms)	0.981 [0.948–0.996]	23	17	−8.0	[−51.4, 35.4]

CCC: Lin’s concordance correlation coefficient; RMSE: root mean square error; MAE: mean absolute error; LoA: limits of agreement; pp: percentage points of the gait cycle.

**Table 4:** Comparison of the present approach with selected deep learning-based IMU gait-event detection studies.

Study	Sensor Location	Study Population	Evaluation Protocol	Tolerance Window	$F_{1}$ Score	Temporal Error
[21]	Waist	healthy adults	LOOCV	NR	HS: 99.51%; TO: 99.28%	MAE: HS 20.43; TO 21.75 ms
[22]	Waist	older adults	Cohort-based train/test	±1–±6 ms	NR	MAE: HS 6.24; TO 5.24 ms
[23]	Shank/ankle	healthy/clinical adults	Independent data split	NR	IC: ≥96%; FC: ≥94%	Median: ≤5 ms; IQR: ≤20 ms
[27]	Bilateral shank	healthy, glaucoma, cLBP	Independent data split	NR	NR	NR
**Ours**	Shank	healthy/NSLBP older adults	Participant-independent five-fold CV	±30 ms	IC/FO: >95%	MAE: <12 ms

NR: not reported in a directly comparable form; LOOCV: leave-one-out cross-validation; CV: cross-validation; IC: initial contact; FC: final contact; HS: heel strike; TO: toe-off; FO: foot-off; MAE: mean absolute error; IQR: interquartile range; cLBP: chronic low back pain; NSLBP: non-specific low back pain.

## Data Availability

The internal data supporting the findings of this study is deposited in Zenodo and available at https://doi.org/10.5281/zenodo.17899477 (accessed 10 August 2026). The source code is available at https://github.com/MS-AI-OVGU/gait_ml (accessed 10 August 2026). The analyses were performed using Python 3.10.18, and the repository includes the package dependencies and version information required to reproduce the analyses. The source code is released under the PolyForm Noncommercial License 1.0.0 for noncommercial research use. The external dataset is publicly available as described in [28].

## Abbreviations

The following abbreviations are used in this manuscript:

**Abbreviation**	**Definition**
CCC	Concordance correlation coefficient
CNN	Convolutional neural network
CSAV	Cumulative mediolateral shank angular velocity
CV	Cross-validation
DL	Deep learning
FO	Foot-off
GRU	Gated recurrent unit
IC	Initial contact
IMU	Inertial measurement unit
LE-GRU	Label-expanded gated recurrent unit
LoA	Limits of agreement
LSTM	Long short-term memory
MAE	Mean absolute error
ML	Machine learning
NE	Non-event
NSLBP	Non-specific low back pain
RMSE	Root mean square error
RNN	Recurrent neural network
TCN	Temporal convolutional network

## Nomenclature

**Symbol**	**Definition**	**Unit**
*X*	Input IMU feature tensor	–
*Y*	Model output tensor for three gait-event classes	–
R	Set of real numbers	–
*B*	Number of signal windows	–
*L*	Sequence length	samples
*D*	Number of IMU input channels	channels
$GRU_{1}$	First gated recurrent unit layer	–
$GRU_{2}$	Second gated recurrent unit layer	–
α	Initial learning rate	–
$t_{\mathrm{IC},i}$	Time of the *i*th initial-contact event	s
$t_{\mathrm{FO},i}$	Time of the *i*th foot-off event	s
$T_{\mathrm{stride},i}$	Stride time of the *i*th gait cycle	s
$P_{\mathrm{stance},i}$	Stance phase of the *i*th gait cycle	%
*i*	Gait-cycle/event index	–
$F_{1}$	F1 score	–
